# Counteracting HPV Cervical and Anal Infection through Dietary Supplementation of EGCG, Folic Acid, Vitamin B12 and Hyaluronic Acid: Clinical Case Reports

**DOI:** 10.3390/jcm13123597

**Published:** 2024-06-19

**Authors:** Marco Calcagno, Bernadette Incocciati, Ludovica Di Fraia, Vittorio Unfer

**Affiliations:** 1 Department of Obstetrics and Gynecology, Santo Spirito Hospital, 00193 Rome, Italy; 2 Di Fraia Laboratori s.r.l., 00034 Colleferro, Italy; 3 A.G.Un.Co. Obstetrics and Gynaecology Center, 00155 Rome, Italy; 4 UniCamillus–Saint Camillus International University of Health Sciences, 00131 Rome, Italy

**Keywords:** HPV, anal infection, cervical infection, persistence, EGCG, folic acid, vitamin B12, hyaluronic acid

## Abstract

**Background:** Human papilloma virus (HPV) infection and the management of its persistence is still a great medical challenge. Recently, scientific evidence has supported the potential therapeutic effects of four combined natural molecules—epigallocatechin gallate (EGCG), folic acid, vitamin B12 and hyaluronic acid (HA)—in counteracting HPV DNA positivity and related cytological lesions. **Methods**: Each patient of these five clinical cases had persistent HPV positivity in the anogenital site and assumed a dietary supplement based on a combination of 200 mg of EGCG, 50 mg of HA, 1 mg of vitamin B12 and 400 mcg of folic acid (Pervistop^®^, Farmares s.r.l., Rome, Italy) at a dosage of 1 or 2 caps/day for 6 or 3 months, respectively, depending on clinical history. **Results**: After treatment, all the patients reported a negative HPV DNA test and improved cytological lesions, thus demonstrating the ability of these combined molecules to counteract both anal and cervical HPV infection and related manifestations. **Conclusions**: Overall, these data corroborate previous evidence about the effectiveness of such natural molecules in the management of HPV infection and its persistence. Naturally, further studies with a larger population and long-term follow-up will contribute to reinforce the positive effects of this dietary supplement in counteracting HPV infection.

## 1. Introduction

Human papilloma virus (HPV) is one of the most common sexually transmitted infections (STIs) around the world [1]. The HPV family includes over 100 types of viruses, and 40 of them may infect the genital tract [2]. According to their oncogenic potential, all the papillomaviruses are classified as low-risk (LR), which usually lead to benign lesions (such as warts, condylomas and recurrent respiratory papillomatosis) or precancerous lesions, and high-risk (HR), which are highly associated with cancers of the lower genital tract, anus and oropharynx both in men and women [3]. The most frequent anogenital cancer is cervical cancer, a major health burden worldwide, particularly in less developed countries. Although several tests can effectively screen for cervical cancer, it remains the fourth most common cancer among women, and the second most common in women between 15 and 45 years of age [4]. 

HPV infection and, in general, STIs may negatively impact patient quality of life, influencing their physical, psychological and sexual health. A recent study reported that symptoms of depression are more common and severe in patients with STIs compared to patients affected by other types of conditions, such as inflammatory bowel disease, and sexual functioning also gets worse in patients with STIs [5].

HPV infections affecting cervical tissue can induce cervical intraepithelial (CIN) lesions of various degrees. Pre-malignant changes represent histological abnormalities ranging from atypical squamous cells of undetermined significance (ASCUS) and low-grade cervical dysplasia (LSIL/CIN1) that could progress to moderate dysplasia (CIN2) or severe dysplasia/carcinoma in situ (CIN3/CIS). Depending on their severity grade, they can spontaneously revert or progress toward tumor development, thus guiding management decisions. While ASCUS/CIN1 need no treatment at the time of diagnosis, as most of them spontaneously regress, high-grade lesions (HSIL/CIN2 or CIN3) may undergo conservative surgical procedures, including ablative methods that destroy the affected cervical tissue [6]. Nevertheless, women with HSIL (CIN2/3) still have an increased risk of developing recurrent CIN2 or worse (CIN2+) after a loop electrosurgical excision procedure (LEEP) [7].

Besides the cervical anatomical site, in the last few years, the prevalence of HPV infections in the anal region has increased as well. Previous studies have demonstrated that in about 93% of cases, HPV infection may correlate with squamous anal carcinoma [8] and, at the same time, about the 88% of anal cancers correlate with HPV positivity [9]. Similarly to cervical region, anal cancer may also progress starting from a preinvasive condition—the anal squamous intraepithelial neoplasia (AIN)—which is the precursor lesion to anal squamous cell carcinoma. As for the cervical site, the Bethesda System terminology defines different grades of AIN: AIN1 corresponds to LSIL, which can have the potential to progress to HSIL, and AIN 2/3 correspond to HSIL [10], which are premalignant lesions that are able to progress to anal cancer, depending on some circumstances [11,12]. However, the percentage of regression and progression of the lesions in these patients is still unclear [10]. The HPV types implicated in anal intraepithelial neoplasia are similar to those described for the cervix, and it is not uncommon to observe an infection by multiple HPV types [13]. In addition, due to the anatomical proximity of the genital and anus area, the presence of cervical HPV infection may represent the strongest risk factor for anal HPV infection [14]. Indeed, women with cervical HPV infection have more than a 3-fold increased risk of concurrent anal infection [15]. Even though every anatomical site has different risk factors for the prevalence of HPV infections, the acquisition of HPV infection in one anatomical site may increase the risk for developing HPV infection in another anatomical site. 

To date, local therapy, such as topical treatments, ablation or surgery, are the most used approaches in the management of anal lesions and cancer. However, a great proportion of patient risks do not respond to the treatment, meaning that the infection may recur. The recurrence of AIN is presumably mediated by ongoing exposure to predisposing risk factors, in particular ongoing HPV infection and its persistence [10]. The latter is a crucial feature of HPV infection [16]. Several definitions may indicate the meaning of persistence [17,18], including one considering persistence as the presence of the same viral HPV genotype in the same patient for more than nine months. Furthermore, a persistent infection represents a high-risk factor for cancer development due to the higher possibility for the integration of viral DNA in the host genome, which leads to neoplastic transformation [19]. From a molecular point of view, once the viral genome has integrated into the host genome, this leads to a breakpoint in the E2 genetic sequence, resulting in the de-repression of E6 and E7 viral oncogenes. The carcinogenic process, which starts with E6 and E7 de-repression, includes the accumulation of additional alterations in the host genome that lead to the invasive cancer phenotype. For instance, E6 and E7 proteins abrogate the function of p53 and pRb proteins, two important tumor suppressors [20,21]. In addition, several factors may contribute to the promotion of HPV persistence in different anatomical sites, like genital co-infections. For instance, female gender, HR-HPV infection, smoking and co-infection with Ureaplasma are all factors statistically associated with the persistence of genital HPV infection [22].

The prevention of HPV infection and related cancers clearly relies on the importance of HPV vaccination and other strategies, such as screening programs aimed at detecting and treating pre-cancerous lesions before they progress to cancer and at potentially reducing the viral HPV load, which is a public health problem. Indeed, the World Health Organization (WHO) recommends including HPV vaccines in national immunization programs considering the prevention of cervical carcinoma and other HPV-associated diseases as a public health priority [23]. A crucial topic is also introducing and sustaining HPV vaccination programs in low- and middle-income countries, in which several type of barriers—government, healthcare providers, society and individuals—need to be addressed and solved [24]. 

Unfortunately, HPV persistence remains an untargeted problem, and new strategies are necessary to address this therapeutic gap. 

Recent scientific evidence has reported that the combination of natural molecules, such as epigallocatechin gallate (EGCG), folic acid, vitamin B12 and hyaluronic acid (HA), may represent a potential synergic treatment for preventing or counteracting HPV persistence [25,26,27,28]. A recently published in vitro study on HPV-positive cervical cancer cells (HeLa cells) highlighted the synergic effect of these molecules in increasing apoptosis by upregulating p53 and downregulating E6/E7 expression, respectively [28]. In particular, treating such HPV-positive cells with a combination of EGCG, folic acid, vitamin B12 and HA had a stronger effect on apoptosis and p53 and E6/E7 expression levels than treatments with the individual molecules. 

In addition, in the last few years, some clinical studies have started to evaluate the oral administration of these natural molecules in counteracting cervical HPV DNA positivity and improving cytological lesions [25,26]. These studies rely on a large body of the literature that strongly supports the activity of each of the selected natural molecules in the HPV field. Indeed, several in vitro studies have demonstrated that EGCG has an antiproliferative activity on cervical cancer cells by interfering with the HPV life cycle and suppressing the E6/E7 oncoproteins responsible for the viral oncogenic activity and cancer development [29,30,31]. Furthermore, a recent study demonstrated the effect of EGCG on stimulating the interferon (IFN) pathway, which is one of the escape mechanisms of HPV, thus reinforcing the innate anti-viral immunity against HPV [32]. In addition, different clinical studies have demonstrated its efficacy, both as a topical and oral formulation, in recovering HPV infections [33,34], although its topical application may expose patients to undesirable effects. The randomized clinical study by Ahn and colleagues revealed a significant improvement in the degree of cervical lesions after 8-/12-week treatments with 200 mg/day of EGCG [34].

Plasmatic levels of both folic acid and vitamin B12 are inversely correlated with homocysteine levels and with an increased risk of cervical cancer; lower serum levels of folate and higher homocysteine levels might increase the susceptibility to cervical cancer [35]. A clinical study demonstrated that in the case of folate deficiency, persistent HPV infection and the progression of cervical dysplasia were increased [36]. Indeed, women with HSILs (HPV+) and LSILs (HPV+) exhibited higher serum levels of homocysteine than the control group, and the more the cervical dysplasia increased, the more the serum homocysteine levels increased, especially in the presence of HPV [37]. Both folic acid and vitamin B12 are also necessary for the synthesis of S-adenosylmethionine (SAM), which acts in various methylation reactions as the main donor of methyl groups [38]. Low levels of folic acid decrease DNA methylation and consequently increase the frequency of fragile sites on DNA that are the same fragile sites that are preferential for HPV16 integration [39]. Therefore, both folate and vitamin B12, by maintaining genomic stability, may prevent the integration of HPV into cervical cells’ genomes, and the higher their levels, the lower the frequency of diagnosis of HR-HPV. Indeed, clinical studies have demonstrated that subjects with higher folate and vitamin B12 status are 73% less likely to test positive for HR-HPV types and are more likely to test negative [40,41]. 

Furthermore, HPV infection is mainly acquired through sexual intercourse and through close skin-to-skin contact, which preferably involves areas undergoing trauma and/or minor injuries. Indeed, losing continuity in the epithelia allows the viral particles to penetrate and infect cells [42]. Therefore, preserving or restoring the integrity of the epithelium may represent one of the goals for preventing HPV infections. Hyaluronic acid (HA), specifically both low- and very-low-weight molecular hyaluronic acid (LMWHA and vLMWHA), promotes the process of wound-healing repair by stimulating the production of pro-inflammatory factors [43,44]. Indeed, as reported in a recent clinical study, HA accelerates the re-epithelization process in human skin wounds compared to pre-treatments with saline solution [45]. Furthermore, another clinical study demonstrated that HA may act not only as a preventive treatment but also as a pivotal adjuvant approach to boost the efficacy of other compounds and decrease the number of lesions. Riemma and colleagues found that a combination of vaginal HA soft gel caps with an oral administration of echinacea extracts seemed to boost viral clearance and reduce the persistence of LSIL/CIN1 lesions during a 12-month follow-up [46]. 

Considering all the reported evidence, some clinical studies have started to turn on a spotlight on the use of EGCG, folic acid, vitamin B12 and HA to target HPV persistence. To date, indeed, the available treatments only target the clinical signs of HPV infection, including condylomas or cervical lesions, but no specific therapies counteract viral persistence. The aim of this paper is to present different clinical cases in which the association of EGCG, folate, vitamin B12 and HA counteract cervical and, for the first time, anal HPV infection in patients with a story of HPV persistence, thus corroborating the evidence about the effectiveness of this approach.

## 2. Materials and Methods

### 2.1. Patients and Oral Dietary Supplementation

All five patients were followed according to the usual clinical practice, in accordance with the Declaration of Helsinki, at the private clinics of Gynecology and Obstetrics of Agunco (Rome, Italy). Written informed consent was obtained from the patients for the publication of the details of their medical cases. All the evaluated patients had a history of persistence of HPV infection, and none of them, neither at the moment of enrolment nor in the previous months, underwent other pharmaceutical or nutraceutical treatments. 

Patients assumed a dietary supplement based on a combination of 200 mg of EGCG, 50 mg of HA, 1 mg of vitamin B12 and 400 mcg of folic acid (Pervistop^®^, Farmares s.r.l., Rome, Italy) at a dosage of 1 or 2 caps/day for 6 or 3 months, respectively, depending on the patients’ clinical history. 

### 2.2. Cervical–Vaginal Cytology (ThinPrep Pap Test)

The examination of cervical–vaginal cytology is primarily a cervical cancer screening test. This test consisted of a procedure that gently removed cells with an endocervical brush and spatula (Cytobrush Plus GT and Pap Perfect Plastic Spatula; Medscad Trumbull, CT) from the surface of the cervix and the area around it. Then, the scraped cervical cells were placed into a ThinPrep Pap test vial containing 20 mL of PreservCyt^®^ Solution (Cytyc Corp., Marlborough, MA, USA) for analysis. The cytological analysis and relative results were classified using the 2001 Bethesda System for reporting cervical cytology [12].

### 2.3. Anoscopy

Anoscopy is an examination of both the anal canal and rectum, which is performed through an anoscope to help in diagnosing anal and rectal conditions. In the anal anatomical region, low-grade lesions are defined as AIN1, while high-grade lesions as AIN2 and AIN3. The system more accurately reflects the biology of these HPV-related lesions [47].

### 2.4. HPV DNA Genotyping Test

A BD Onclarity^TM^ HPV Assay (BD, Franklin Lakes, NJ, USA) was used to detect 14 HR-HPV genotypes, providing the capability of extended genotyping through the individual detection of HPV 31, 51 and 52 (in addition to 16, 18 and 45) and the pooled detection of 33/58 (P1), 56/59/66 (P2) and 35/39/68 (P3). An endogenous human beta-globin sequence was detected as a sample validity control, for sample extraction and for amplification efficiency [48,49]. 

All the samples were tested following the manufacturer’s instructions. Briefly, 0.5 mL of each cervical sample was added to an LBC tube (a suitable solution produced by BD) to reach a final volume of 2.2 mL. From this solution, 0.8 mL of the sample was automatically taken by the instrument to perform nucleic acid extraction using the extraction chemistry developed by BD (BD FOX ™). The extracted DNA was then eluted to a final volume of 400 microliters, and 50 microliters were automatically pipetted into each of the three wells containing the dried master mix to perform real-time PCR. An algorithm verified the adequacy of the sample using the amplification of the human beta-globin gene. Validation of the analysis of the HPV DNA test was guaranteed by the BD company itself that provided the device for the analysis. 

### 2.5. HPV mRNA Test

An APTIMA HPV Assay was used as a qualitative test based on the direct detection of the expression of E6 and E7 mRNA oncoproteins from the 14 types of HR-HPV (16, 18, 31, 33, 35, 39, 45, 51, 52, 56, 58, 59, 66 and 68) through real-time amplification (48, 49). Validation of the analysis of the HPV mRNA test was guaranteed by the Hologic company itself that provided the device for the analysis. 

## 3. Results

### 3.1. Patient 1

A 39-year-old woman came to the clinics of Gynecology and Obstetrics in January 2023 due to a three-year history of HPV persistence. In 2019, a first proctological examination reported two whitish formations suspicious for condylomatosis, which were subsequently surgically removed, thus revealing AIN1 lesion through the histological exam. 

In April 2020, a rectal HPV DNA genotyping test revealed the first positivity of the LR-HPV 61 genotype. After one year, in February 2021, the follow-up control indicated the persistence of the infection in the anal anatomic site due to the same LR-HPV 61 genotype. Two years later, in January 2023, an anoscopy identified a condylomatous lesion at a size of 5 mm with surface mosaicism that was positive with 5% acetic acid. At the same time, the patient still exhibited LR-HPV 61 genotype positivity in the anal region. Therefore, she started taking an oral dietary supplement based on EGCG, folic acid, vitamin B12 and HA at a dosage of 2 cps/day for 3 months. In June 2023, the 5 mm condylomatous lesion was burnt, and the patient repeated the rectal HPV DNA test. After the 3-month treatment with the dietary supplement, the results of the test revealed, for the first time after a three-year history of HPV persistence, a negative result for HPV genotypes in the anal region, including LR-HPV 61, thus suggesting the effectiveness of the dietary supplementation in treating persistent HPV anal infection. 

### 3.2. Patient 2

A 48-year-old woman with a one-year history of HPV persistence came to the clinics of Gynecology and Obstetrics in June 2023 due to a follow-up visit. The patient had been exhibiting positivity to cervical LR-HPV 81 for over one year. In June 2023, the patient repeated a ThinPrep Pap test with an HPV DNA genotyping test as well. The cytological analysis performed on the endo- and exocervical material revealed neither intraepithelial nor malignant lesions but only unspecific flogosis and hyperkeratosis. Instead, the molecular DNA analysis reported cervical positivity to the LR-HPV 81 genotype, thus confirming the persistence of the viral cervical infection. Therefore, the patient started taking an oral dietary supplement based on EGCG, folic acid, vitamin B12 and HA at a dosage of 1 cps/day for 6 months. The following ThinPrep Pap test with an HPV DNA genotyping test, both performed 6 months after in December 2023, revealed an improved medical condition. In particular, the cytological analysis confirmed the absence of intraepithelial or malignant lesions along with the recovery of both flogosis and hyperkeratosis. Moreover, the HPV DNA genotyping test revealed negativity to HPV genotypes, including LR-HPV 81, thus resolving the HPV infection after more than one year of persistence. 

### 3.3. Patient 3

A 27-year-old woman came to the clinics of Gynecology and Obstetrics in June 2023. The first colposcopy analysis performed one year earlier, in 2022, reported a cervical mucosa with LSIL/CIN1 squamous intraepithelial lesion and focal regions with HSIL/CIN2. In January 2023, an HPV DNA genotyping test revealed the presence of HR-HPV 56 in the cervical anatomical region, confirming the persistence of the same genotype that had already been found for the first time the year before. After 6 months, she repeated the cervical HPV DNA genotyping test, and the results confirmed the positivity of the same HR-HPV 56 genotype. At that moment, in June 2023, she started taking a dietary supplement based on EGCG, folic acid, vitamin B12 and HA at a dosage of 1 cps/day for 6 months. After this period, she underwent both a ThinPrep Pap test and HPV DNA genotyping analysis. The first test revealed neither malignant cells nor intraepithelial lesions, thus demonstrating an improvement over the previous clinical picture of LSIL/CIN1 with focal regions of HSIL/CIN2. Moreover, the DNA test reported the absence of the investigated HPV genotypes, including HR-HPV 56, thus highlighting the negativity in the HPV DNA and finally resolving the persistent infection. 

### 3.4. Patient 4

A 35-year-old woman came to the clinics of Gynecology and Obstetrics for a gynaecological control visit in July 2023. A ThinPrep Pap test analysis performed on cervical cells revealed the presence of atypical cells of an uncertain significance, known as ASCUS. Along with this, the medical report also indicated intense flogosis and hyperkeratosis. A concomitant HPV DNA genotyping cervical test reported the presence of HR-HPV 39, confirming the persistence of the same genotype that had already been found for the first time the previous year. At that moment, the patient started taking a dietary supplementation of EGCG, folic acid, vitamin B12 and HA at a dosage of 1 cps/day for 6 months. After this period, she repeated the HPV DNA genotyping test, and the result was completely negative in terms of the different evaluated HPV genotypes, including HR-HPV 39, thus highlighting the counteracting of the one-year-persistent HPV infection. 

### 3.5. Patient 5

A 37-year-old woman came to the clinics of Gynecology and Obstetrics in February 2023 due to a seven-year history of HPV persistence. In 2015, because of a sensation of vaginal itching that she had been experiencing for a year, she underwent a colposcopy and, concomitantly, a ThinPrep Pap test. The Schiller test was absorbent, the squamous-columnar junction was visible and the epithelium appeared physiologically trophic. Instead, the physician indicated a region of abnormal transformation of a low grade (grade I) with a thin white epithelium. The concomitant medical report of the ThinPrep Pap test reported a diagnosis of LSIL/HPV. 

One year later, in 2016, the patient underwent a control ThinPrep Pap test with a HPV DNA genotyping test, and the medical report indicated the presence of HR-HPV 18. In the following months, she underwent another colposcopy that revealed a physiological squamous-columnar junction with a trophic epithelium and an abnormal region with a thin white epithelium and glandular thickened outlets, and she underwent a treatment with laser CO_2_. 

In 2020, another cervical HPV DNA genotyping test confirmed a persistent infection due to HR-HPV 18, while the cytological analysis reported neither intraepithelial lesions nor malignancy with only evidence of dyskeratosis. 

In 2022, she underwent two colposcopies and another laser CO_2_ treatment, and in 2023, she repeated the cervical DNA genotyping test, which still reported the presence of the HR-HPV 18 genotype. Later, in February 2023, she started taking an oral supplementation of EGCG, folic acid, vitamin B12 and HA at a dosage of 1 cps/day for 6 months. After this period, she underwent a series of follow-up controls. A ThinPrep Pap exam confirmed the absence of both intraepithelial lesions and malignancy; a cervical HPV DNA genotyping test was, for the first time, negative, and an mRNA test for HPV was also negative after seven years of persistent infection.

## 4. Discussion

These clinical cases corroborated the effectiveness of the oral combination of four natural molecules—EGCG, vitamin B12, folic acid and HA—in counteracting positivity to HPV infection in both anal and cervical regions (Table 1).

So far, no medical tools have been effective in managing HPV persistence, since an effective treatment against the clearance of HPV is still lacking [50]. General guidelines only advise monitoring patients with low-grade lesions through recurrent controls until they spontaneously clear the infection or develop cervical or anal neoplasia [51]. Instead, in cases of more serious lesions, surgical procedures are the best practice, even though they may not guarantee the clearance of the virus and the recurrence of new lesions [52]. 

In addition, in cases of anal infection, despite its high frequency among healthy and sexually active individuals and its correlation with anal squamous cell carcinoma, AIN is frequently underappreciated by healthcare providers. For this reason, it is often detected in advanced stages [10], and its clinical management may be difficult for low response rates or for the problem of recurrence, in which HPV persistence plays a central role. Therefore, all this evidence strongly corroborates the existing gap in clinical practice and in the management of HPV infection for counteracting its persistence [53,54]. 

Furthermore, chronic infections may often meet the problem of self-medication, which is an important public health issue with a varied prevalence across the world [55,56]. The use of drugs to treat self-diagnosed symptoms or the incorrect use of prescribed drugs—which the WHO has defined as self-medication—may have serious negative consequences for general health. In addition, the irrational use of antimicrobials may lead to delaying appropriate treatments, pathogen resistance and increased morbidity [57]. 

A common feature among the different clinical backgrounds of the evaluated five patients was the persistence of HPV infection, which had been experienced for times ranging from 1 to 7 years with the occurrence of cytological lesions of different grades. Considering this target, a natural supplement that is able to counteract HPV persistence becomes highly important in clinical practice.

Before obtaining the first evidence about the combination of these four natural molecules, the available studies pointed out the efficacy of each single molecule against viral infection, as follows: EGCG as an antiproliferative and proapoptotic factor [29,30,32,34,58]; vitamin B12 and folic acid as methylation agents that are able to block viral proliferation and persistence [59]; and HA as a physical barrier that restores the integrity of the epithelium [44], thus preventing HPV infection. However, in vitro evidence has only recently demonstrated the synergic effect of the combination of EGCG, folic acid, vitamin B12 and HA in counteracting HPV infection by increasing apoptosis and p53 expression in HPV-infected cervical cells [28]. 

Four of the reported clinical cases with positivity to cervical HPV DNA test and with cytological lesions of different grades restored the negativity to viral DNA and improved the clinical cytological conditions and related flogosis after 6 months of dietary supplementation. It is noteworthy that one patient with HPV anal positivity became negative after 3 months of taking the dietary supplement at the dosage of 2 cps/day, thus also revealing, for the first time, the effectiveness of these oral combined natural molecules in an anatomical region different from the cervical one. 

The obtained data agree with previous clinical evidence—summarized in Table 2—supporting the interesting effects of such natural molecules in the HPV field by blocking the progression of intraepithelial lesions and reducing the HPV DNA viral load [25,26]. In particular, a previous case report has revealed, for the first time, the effectiveness of such a combination in improving cytological lesions in a young patient with 9-year HPV persistence, thus preventing or delaying a hysterectomy surgery [25]. Furthermore, a pilot clinical study by Aragona and colleagues highlighted that 17 out of 20 women treated with combined EGCG, folic acid, vitamin B12 and HA achieved a full viral clearance and showed no cytological or histological evidence of lesions following the treatment. In this way, the authors turned on a spotlight on the use of these natural molecules to improve HPV clinical manifestations and its persistence [26]. 

Overall, these five clinical cases corroborated previous data about the protective and therapeutic effects of EGCG, folic acid, vitamin B12 and HA, acting on different levels of HPV infection. In addition, for the first time, they reported preliminary data on the efficacy of such natural molecules toward anal HPV infection, thus also guiding clinical practice toward the use of such molecules. 

Naturally, the limited number of the clinical cases and the absence of a long-term follow-up after treatment represent the main limitations of the obtained results. For this reason, further and longer-term studies with a larger population and with the evaluation of different anatomical sites (such as the anal and oral regions) are necessary to strongly corroborate the effectiveness of this dietary supplement and to extend its promising use in the management of HPV persistence. 

## 5. Conclusions

The management of HPV infection and its persistence remains a critical topic in clinical practice. These five clinical cases evidenced the positive effects of the above-mentioned combined natural molecules—EGCG, folic acid, vitamin B12 and HA—to treat HPV DNA positivity both at the anal and cervical regions. These data are in line with previous preclinical and clinical evidence supporting the clinical use of such dietary supplements to counteract HPV persistence. Indeed, to date, physicians have vaccines and screening programs for preventing infections and local approaches for targeting manifestations such as cervical lesions and condylomas, but the persistence of the infection still remains an unsolved problem. 

The obtained results corroborate the recent existing evidence for the clinical use of these natural molecules, extending their efficacy also to the anal region. In this way, they further support the possibility of considering this dietary supplement as an effective and promising approach in the management of HPV infection and its persistence. Naturally, future clinical studies designed with a larger population and longer-term follow up that also consider HPV infection in other anatomical regions, will help to reinforce and extend the clinical use of these natural molecules in the management of HPV. 

## Figures and Tables

**Table 1:** Schematic representation of five clinical case reports.

Patient	Age	HPV Genotype and Cytological Profile	Anatomical Site	Persistence	Treatment	Outcomes
Patient 1	39	LR-HPV 61 and condylomatous lesion of a size of 5 mm	anal	3 years	2 cps/day 3 months	Negative to HPV DNA anal test after 3 years of persistence.
Patient 2	48	LR-HPV 81 and unspecific flogosis and hyperkeratosis	cervical	1 year	1 cps/day 6 months	Negative to HPV DNA cervical test after 1 year of persistence; restored flogosis and hyperkeratosis.
Patient 3	27	HR-HPV 56 and LSIL/CIN1 with focal regions of HSIL/CIN2	cervical	1 year	1 cps/day 6 months	Negative to HPV DNA cervical test after 1 year of persistence; restored intraepithelial lesions.
Patient 4	35	HR-HPV 39 and ASCUS, intense flogosis and hyperkeratosis	cervical	1 year	1 cps/day 6 months	Negative to HPV DNA cervical test after 1 year of persistence.
Patient 5	37	HR-HPV 18 and LSIL/HPV	cervical	7 years	1 cps/day 6 months	Negative to HPV DNA and mRNA cervical test after 7 years of persistence; absence of intraepithelial lesions or malignancy.

The table schematically represents the principal clinical features of the five patients: age, low-risk (LR) or high-risk (HR) genotype, involved anatomical region, years of persistence, treatment and results.

**Table 2:** Schematic representation of case reports or studies in the literature supporting the beneficial effects of reported natural molecules against HPV infection.

Authors	Year	Type of Study	Methods	Main Findings
Grandi et al. [25]	2023	Case report	9 years of HR-HPV 16 persistence and cervical lesions; 2 cps/day for 8 weeks	No more cervical lesions after treatment, thus avoiding an invasive hysterectomy surgery.
Laganà et al. [27]	2023	Review	-	Evidence supporting the anti-viral effect of each natural molecule (EGCG, FA, vitamin B12 and HA).
Frega et al. [28]	2023	In vitro study	Evaluation of a combination of EGCG, FA, vitamin B12 and HA on HPV-positive cervical cancer cells (HeLa).	Evidence for the synergic effect of EGCG, FA, vitamin B12 and HA in counteracting HPV infection by increasing p53 and apoptosis.
Aragona et al. [26]	2023	Pilot clinical study	Forty patients with persistent HPV infection and cervical lesions. Treated group received 1 cps/day for 12 weeks; control group received no treatment.	After treatment, 17 of 20 women achieved a full viral clearance without having cytological cervical lesions.

The table schematically represents principal evidence in the literature supporting the use of such combined molecules in counteracting HPV infection.

## Data Availability

Data will be made available upon reasonable request.

## References

[1] Kombe Kombe A.J., Li B., Zahid A., Mengist H.M., Bounda G.A., Zhou Y., Jin T. (2020). Epidemiology and Burden of Human Papillomavirus and Related Diseases, Molecular Pathogenesis, and Vaccine Evaluation. Front. Public. Health.

[2] Van Doorslaer K. (2022). Revisiting Papillomavirus Taxonomy: A Proposal for Updating the Current Classification in Line with Evolutionary Evidence. Viruses.

[3] Schiffman M., Doorbar J., Wentzensen N., de Sanjosé S., Fakhry C., Monk B.J., Stanley M.A., Franceschi S. (2016). Carcinogenic human papillomavirus infection. Nat. Rev. Dis. Primers.

[4] Sung H., Ferlay J., Siegel R.L., Laversanne M., Soerjomataram I., Jemal A., Bray F. (2021). Global Cancer Statistics 2020: GLOBOCAN Estimates of Incidence and Mortality Worldwide for 36 Cancers in 185 Countries. CA Cancer J. Clin..

[5] Ciccarese G., Drago F., Copello F., Bodini G., Rebora A., Parodi A. (2021). Study on the impact of sexually transmitted infections on Quality of Life, mood and sexual function. Ital. J. Dermatol. Venerol..

[6] World Health Organization (2021). WH Guideline for Screening and Treatment of Cervical Pre-Cancer Lesions for Cervical Cancer Prevention. WHO Guidelines Approved by the Guidelines Review Committee.

[7] Ding T., Li L., Duan R., Chen Y., Yang B., Xi M. (2023). Risk factors analysis of recurrent disease after treatment with a loop electrosurgical excision procedure for high-grade cervical intraepithelial neoplasia. Int. J. Gynaecol. Obstet..

[8] Carvalho N.S., Ferreira A.M., Bueno C.C. (2011). HPV infection and intraepithelial lesions from the anal region: How to diagnose?. Braz. J. Infect. Dis..

[9] Benevolo M., Latini A., Rollo F., Giuliani M., Giglio A., Giuliani E., Cristaudo A., Morrone A., Dona M.G. (2023). Incidence of abnormal anal cytology in HIV-infected and HIV-uninfected men who have sex with men. Cancer Cytopathol..

[10] Roberts J.R., Siekas L.L., Kaz A.M. (2017). Anal intraepithelial neoplasia: A review of diagnosis and management. World J. Gastrointest. Oncol..

[11] Fenger C., Nielsen V.T. (1986). Intraepithelial neoplasia in the anal canal. The appearance and relation to genital neoplasia. Acta Pathol. Microbiol. Immunol. Scand. A.

[12] Solomon D., Davey D., Kurman R., Moriarty A., O’Connor D., Prey M., Raab S., Sherman M., Wilbur D., Wright T. (2002). The 2001 Bethesda System: Terminology for reporting results of cervical cytology. JAMA.

[13] Ramamoorthy S., Liu Y.T., Luo L., Miyai K., Lu Q., Carethers J.M. (2010). Detection of multiple human papillomavirus genotypes in anal carcinoma. Infect. Agent. Cancer.

[14] Dzundova M.N., Sehnal B., Zikan M., Kocian R., Dubova O., Hubka P., Dostalek L., Kabele P., Brtnicky T., Slama J. (2023). Risk Factors for the Anal and Oral Human Papillomavirus (HPV) Infections among Women with Severe Cervical Lesions: A Prospective Case-Control Study. Biomedicines.

[15] Hernandez B.Y., McDuffie K., Zhu X., Wilkens L.R., Killeen J., Kessel B., Wakabayashi M.T., Bertram C.C., Easa D., Ning L. (2005). Anal human papillomavirus infection in women and its relationship with cervical infection. Cancer Epidemiol. Biomark. Prev..

[16] Shanmugasundaram S., You J. (2017). Targeting Persistent Human Papillomavirus Infection. Viruses.

[17] Giuliano A.R., Harris R., Sedjo R.L., Baldwin S., Roe D., Papenfuss M.R., Abrahamsen M., Inserra P., Olvera S., Hatch K. (2002). Incidence, prevalence, and clearance of type-specific human papillomavirus infections: The Young Women’s Health Study. J. Infect. Dis..

[18] Moscicki A.B., Ellenberg J.H., Crowley-Nowick P., Darragh T.M., Xu J., Fahrat S. (2004). Risk of high-grade squamous intraepithelial lesion in HIV-infected adolescents. J. Infect. Dis..

[19] de Sanjosé S., Brotons M., Pavón M.A. (2018). The natural history of human papillomavirus infection. Best. Pract. Res. Clin. Obstet. Gynaecol..

[20] Travé G., Zanier K. (2016). HPV-mediated inactivation of tumor suppressor p53. Cell Cycle.

[21] Münger K., Scheffner M., Huibregtse J.M., Howley P.M. (1992). Interactions of HPV E6 and E7 oncoproteins with tumour suppressor gene products. Cancer Surv..

[22] Ciccarese G., Herzum A., Pastorino A., Dezzana M., Casazza S., Mavilia M.G., Copello F., Parodi A., Drago F. (2021). Prevalence of genital HPV infection in STI and healthy populations and risk factors for viral persistence. Eur. J. Clin. Microbiol. Infect. Dis..

[23] Aggarwal S., Agarwal P., Gupta N. (2024). A comprehensive narrative review of challenges and facilitators in the implementation of various HPV vaccination program worldwide. Cancer Med..

[24] Ver A.T., Notarte K.I., Velasco J.V., Buac K.M., Nazareno J., Lozanes J.A., Antonio D., Bacorro W. (2021). A systematic review of the barriers to implementing human papillomavirus vaccination programs in low- and middle-income countries in the Asia-Pacific. Asia Pac. J. Clin. Oncol..

[25] Grandi G., Botticelli L., Fraia P.D., Babalini C., Masini M., Unfer V. (2023). The Association of Four Natural Molecules-EGCG, Folic Acid, Vitamin B12, and HA-To Counteract HPV Cervical Lesions: A Case Report. J. Pers. Med..

[26] Aragona C., Bezerra Espinola M.S., Bilotta G., Porcaro G., Calcagno M. (2023). Evaluating the Efficacy of Pervistop((R)), a New Combination Based on EGCG, Folic Acid, Vitamin B12 and Hyaluronic Acid on Patients with Human Papilloma Virus (HPV) Persistent Infections and Cervical Lesions: A Pilot Study. J. Clin. Med..

[27] Laganà A.S., Chiantera V., Gerli S., Proietti S., Lepore E., Unfer V., Carugno J., Favilli A. (2023). Preventing Persistence of HPV Infection with Natural Molecules. Pathogens.

[28] Frega A., Gentili C., Proietti S., Lepore E., Unfer V., Fuso A. (2023). Epigallocatechin gallate, folic acid, vitamin B12, and hyaluronic acid significantly increase apoptosis and p53 expression in HeLa cells. Eur. Rev. Med. Pharmacol. Sci..

[29] Yap J.K.W., Kehoe S.T., Woodman C.B.J., Dawson C.W. (2021). The Major Constituent of Green Tea, Epigallocatechin-3-Gallate (EGCG), Inhibits the Growth of HPV18-Infected Keratinocytes by Stimulating Proteasomal Turnover of the E6 and E7 Oncoproteins. Pathogens.

[30] Singh M., Singh R., Bhui K., Tyagi S., Mahmood Z., Shukla Y. (2011). Tea polyphenols induce apoptosis through mitochondrial pathway and by inhibiting nuclear factor-kappaB and Akt activation in human cervical cancer cells. Oncol. Res..

[31] Al-Hazzani A.A., Alshatwi A.A. (2011). Catechin hydrate inhibits proliferation and mediates apoptosis of SiHa human cervical cancer cells. Food Chem. Toxicol..

[32] Song J.Y., Han J.H., Song Y., Lee J.H., Choi S.Y., Park Y.M. (2021). Epigallocatechin-3-gallate Can Prevent Type 2 Human Papillomavirus E7 from Suppressing Interferon-Stimulated Genes. Int. J. Mol. Sci..

[33] Tatti S., Stockfleth E., Beutner K.R., Tawfik H., Elsasser U., Weyrauch P., Mescheder A. (2010). Polyphenon E: A new treatment for external anogenital warts. Br. J. Dermatol..

[34] Ahn W.S., Yoo J., Huh S.W., Kim C.K., Lee J.M., Namkoong S.E., Bae S.M., Lee I.P. (2003). Protective effects of green tea extracts (polyphenon E and EGCG) on human cervical lesions. Eur. J. Cancer Prev..

[35] Weinstein S.J., Ziegler R.G., Selhub J., Fears T.R., Strickler H.D., Brinton L.A., Hamman R.F., Levine R.S., Mallin K., Stolley P.D. (2001). Elevated serum homocysteine levels and increased risk of invasive cervical cancer in US women. Cancer Causes Control.

[36] Piyathilake C.J., Macaluso M., Chambers M.M., Badiga S., Siddiqui N.R., Bell W.C., Edberg J.C., Partridge E.E., Alvarez R.D., Johanning G.L. (2014). Folate and vitamin B12 may play a critical role in lowering the HPV 16 methylation-associated risk of developing higher grades of CIN. Cancer Prev. Res..

[37] Abike F., Engin A.B., Dunder I., Tapisiz O.L., Aslan C., Kutluay L. (2011). Human papilloma virus persistence and neopterin, folate and homocysteine levels in cervical dysplasias. Arch. Gynecol. Obstet..

[38] Bailey L.B., Gregory J.F. (1999). Folate metabolism and requirements. J. Nutr..

[39] Herbert V. (1986). The role of vitamin B12 and folate in carcinogenesis. Adv. Exp. Med. Biol..

[40] Piyathilake C.J., Henao O.L., Macaluso M., Cornwell P.E., Meleth S., Heimburger D.C., Partridge E.E. (2004). Folate is associated with the natural history of high-risk human papillomaviruses. Cancer Res..

[41] Piyathilake C.J., Badiga S., Paul P., Vijayaraghavan K., Vedantham H., Sudula M., Sowjanya P., Ramakrishna G., Shah K.V., Partridge E.E. (2010). Indian women with higher serum concentrations of folate and vitamin B12 are significantly less likely to be infected with carcinogenic or high-risk (HR) types of human papillomaviruses (HPVs). Int. J. Womens Health.

[42] Woodman C.B., Collins S.I., Young L.S. (2007). The natural history of cervical HPV infection: Unresolved issues. Nat. Rev. Cancer.

[43] Yang H., Song L., Zou Y., Sun D., Wang L., Yu Z., Guo J. (2021). Role of Hyaluronic Acids and Potential as Regenerative Biomaterials in Wound Healing. ACS Appl. Bio Mater..

[44] Gao F., Yang C.X., Mo W., Liu Y.W., He Y.Q. (2008). Hyaluronan oligosaccharides are potential stimulators to angiogenesis via RHAMM mediated signal pathway in wound healing. Clin. Investig. Med..

[45] Nyman E., Henricson J., Ghafouri B., Anderson C.D., Kratz G. (2019). Hyaluronic Acid Accelerates Re-epithelialization and Alters Protein Expression in a Human Wound Model. Plast. Reconstr. Surg. Glob. Open.

[46] Riemma G., Schettino M.T., Munno G.M., Fasulo D.D., Sandullo L., Amabile E., La Verde M., Torella M. (2022). Echinacea angustifolia and Echinacea purpurea Supplementation Combined with Vaginal Hyaluronic Acid to Boost the Remission of Cervical Low-Grade Squamous Intraepithelial Lesions (L-SILs): A Randomized Controlled Trial. Medicina.

[47] Darragh T.M., Colgan T.J., Thomas Cox J., Heller D.S., Henry M.R., Luff R.D., McCalmont T., Nayar R., Palefsky J.M., Stoler M.H. (2013). The Lower Anogenital Squamous Terminology Standardization project for HPV-associated lesions: Background and consensus recommendations from the College of American Pathologists and the American Society for Colposcopy and Cervical Pathology. Int. J. Gynecol. Pathol..

[48] Martinelli M., Giubbi C., Sechi I., Bottari F., Iacobone A.D., Musumeci R., Perdoni F., Muresu N., Piana A., Fruscio R. (2022). Evaluation of BD Onclarity™ HPV Assay on Self-Collected Vaginal and First-Void Urine Samples as Compared to Clinician-Collected Cervical Samples: A Pilot Study. Diagnostics.

[49] Ejegod D.M., Junge J., Franzmann M., Kirschner B., Bottari F., Sideri M., Sandri M.T., Bonde J. (2016). Clinical and analytical performance of the BD Onclarity™ HPV assay for detection of CIN2+ lesions on SurePath samples. Papillomavirus Res..

[50] Xiong Y., Cui L., Bian C., Zhao X., Wang X. (2020). Clearance of human papillomavirus infection in patients with cervical intraepithelial neoplasia: A systemic review and meta-analysis. Medicine.

[51] Boardman L.A., Kennedy C.M. (2008). Management of atypical squamous cells, low-grade squamous intraepithelial lesions, and cervical intraepithelial neoplasia 1. Obstet. Gynecol. Clin. N. Am..

[52] Jancar N., Rakar S., Poljak M., Fujs K., Kocjan B.J., Vrtacnik-Bokal E. (2006). Efficiency of three surgical procedures in eliminating high-risk human papillomavirus infection in women with precancerous cervical lesions. Eur. J. Gynaecol. Oncol..

[53] Bogani G., Di Donato V., Sopracordevole F., Ciavattini A., Ghelardi A., Lopez S., Simoncini T., Plotti F., Casarin J., Serati M. (2020). Recurrence rate after loop electrosurgical excision procedure (LEEP) and laser Conization: A 5-year follow-up study. Gynecol. Oncol..

[54] Ouh Y.T., Cho H.W., Kim S.M., Min K.J., Lee S.H., Song J.Y., Lee J.K., Lee N.W., Hong J.H. (2020). Risk factors for type-specific persistence of high-risk human papillomavirus and residual/recurrent cervical intraepithelial neoplasia after surgical treatment. Obstet. Gynecol. Sci..

[55] Rathod P., Sharma S., Ukey U., Sonpimpale B., Ughade S., Narlawar U., Gaikwad S., Nair P., Masram P., Pandey S. (2023). Prevalence, Pattern, and Reasons for Self-Medication: A Community-Based Cross-Sectional Study From Central India. Cureus.

[56] Alwhaibi M., Malik S.B., Alswailem L., Alruthia Y. (2023). Self-medication among adults with chronic health conditions: A population-based cross-sectional survey in Saudi Arabia. BMJ Open.

[57] Bennadi D. (2013). Self-medication: A current challenge. J. Basic. Clin. Pharm..

[58] Zou C., Liu H., Feugang J.M., Hao Z., Chow H.H., Garcia F. (2010). Green tea compound in chemoprevention of cervical cancer. Int. J. Gynecol. Cancer.

[59] Xiao S., Tang Y.S., Kusumanchi P., Stabler S.P., Zhang Y., Antony A.C. (2018). Folate Deficiency Facilitates Genomic Integration of Human Papillomavirus Type 16 DNA In Vivo in a Novel Mouse Model for Rapid Oncogenic Transformation of Human Keratinocytes. J. Nutr..

